# Immunological mechanisms underlying fibrotic diseases via single-cell technologies

**DOI:** 10.3389/fimmu.2025.1720047

**Published:** 2026-01-09

**Authors:** Yuzo Koda, Nobuhiro Nakamoto, Takanori Kanai

**Affiliations:** 1 Division of Gastroenterology and Hepatology, Department of Internal Medicine, Keio University School of Medicine, Tokyo, Japan; 2 Oncology and Immunology Unit, Research Division, Tanabe Pharma Corporation, Kanagawa, Japan; 3 Japan Agency for Medical Research and Development (AMED), Tokyo, Japan

**Keywords:** fibrosis, immune cells, single cell analysis, single-cell transcriptome, spatial transcriptome

## Abstract

Fibrosis is an organ dysfunction caused by excessive deposition of fibrous components produced by parenchymal cells. Effective treatments are lacking for this progressive pathological condition that manifests in various organs and can lead to mortality. The involvement of the immune system in various aspects of fibrosis development, including chronic organ damage induced by macrophages and T cells, wound healing by macrophages and growth factors such as TGF-β, and polarization toward the type II cytokine phenotype, has been widely reported. Recently, immune cells were also reported to contribute to the resolution phase of fibrotic conditions, highlighting the relevance of immune cell analysis in the understanding of both progression and recovery of fibrotic pathologies. However, owing to the complexity and diversity of disease progression, conventional pathological analyses that focus on individual cells and factors have limitations. Technological advancements, such as next-generation sequencing and spatial transcriptome analysis, have enabled pathological analysis at the single-cell level rendering positional information on cells within organs. These advancements have allowed for the redefinition of heterogeneous cells present in organs and a precise understanding of individual cell phenotypes from small human patient samples. Furthermore, integration of transcriptome, proteome, and epigenome data from each cell has begun to reveal important cell-cell interactions under various fibrotic conditions. This review comprehensively discusses the involvement of the immune system and fibrosis, as well as the key interactions between immune and parenchymal cells unraveled via recent advancements in single-cell analysis of fibrotic pathologies in various organs. It also introduces novel strategies leveraging the latest single-cell analyses for fibrosis treatment.

## 1 Introduction

Fibrosis is a complex and chronic pathological condition in which the parenchymal and immune cells are intricately involved (1). Although fibrosis is directly involved in organ failure in the advanced stages of many chronic immune and inflammatory diseases and is highly lethal, it remains an unmet medical need, as existing therapies often fail to control it. Therefore, elucidating the pathogenic mechanisms involved is crucial for the development of novel treatments. In fibrotic pathology, persistent inflammation and aberrant immune responses driven by myeloid cells, such as macrophages and neutrophils, diverse T-cell subsets, B cells/plasma cells, and innate lymphoid cells, have been reported across various organs (1). In particular, interactions between fibroblasts, which produce the extracellular matrix (ECM) and directly induce organ fibrosis, and immune cells have been suggested as crucial mechanisms in fibrotic disease (2). In fibrotic conditions, fibroblasts are traditionally viewed as passive cells that, upon stimulation (e.g., by macrophage-derived factors), produce ECM (3). However, recent findings indicate that fibroblasts also actively shape the pathological niche, for example, by producing chemokines that recruit leukocytes to the tissue (4). Although immune cell–fibroblast interactions have long been a central theme in fibrosis research, an integrated understanding of fibrotic pathology and intercellular crosstalk is impossible based on disparate low-resolution studies examining specific immune cells in isolation. Moreover, in organ fibrosis, principal effector cells, such as fibroblasts and myeloid cells, often cannot be defined by distinct surface markers, which limits phenotypic characterization and functional validation via cell depletion.

In recent years, the development of next-generation sequencers has driven dramatic advances in single-cell technologies, such as single-cell RNA sequencing (scRNA-seq), single-nucleus RNA-seq, and spatial transcriptomics (1, 5, 6). These single-cell approaches have become powerful tools in fibrosis research to achieve a comprehensive understanding of cell–cell interactions and to redefine cell subsets. By decoding gene expression profiles at the single-cell level, cell subsets that were previously obscured in conventional bulk RNA-seq or surface marker-based FACS analysis have been redefined. The heterogeneity of fibroblasts and immune cells in healthy and diseased states, as well as organ-specific immune cell infiltration patterns and fibroblast alterations, are being rapidly elucidated. Spatial transcriptomic analysis also allows the examination of location-specific cell–cell interactions and pathogenic mechanisms. This approach has greatly contributed to obtaining unbiased insights into the role of each cell and intercellular interactions in fibrotic niches. Furthermore, even in human disease analysis, where limited sample quantity is a major constraint, single-cell techniques enable comprehensive data collection from small amounts of blood or tissue, dramatically improving our understanding of pathogenesis. In addition, human cell-derived organoid models have been developed to study fibrotic diseases. These models enable the use of human cells in a controlled three-dimensional microenvironment and allow for genetic and pharmacological manipulations (7, 8). The combination of single-cell analysis and organoid models may lead to a more detailed understanding of the pathogenesis of fibrosis.

In this review, we provide an overview of common fibrosis mechanisms mediated by the immune system in major organs (liver, lungs, skin, kidneys, and intestine) and summarize the latest insights obtained via single-cell technologies in these fibrotic diseases. We also introduce new therapeutic concepts that have emerged from these findings.

## 2 Liver fibrosis

### 2.1 Background and general mechanisms of liver fibrosis

Globally, liver diseases cause an estimated two million deaths per year (approximately 4% of all deaths) (9). The liver can develop fibrosis due to various chronic liver diseases, such as viral hepatitis, alcoholic hepatitis, metabolic dysfunction-associated steatohepatitis (MASH, formerly NASH), primary sclerosing cholangitis (PSC), primary biliary cholangitis (PBC), and autoimmune hepatitis (AIH). Advanced fibrotic pathology of the liver is known as cirrhosis (10, 11). Liver fibrosis arises from the accumulation of excessive ECM produced by activated myofibroblasts, which originate primarily from hepatic stellate cells and portal fibroblasts in the liver (10). Liver fibrosis is commonly assessed using the standardized pathological scoring of biopsy samples. However, scoring systems, such as FIB-4 and APRI, are also used (12, 13). In addition, evaluations using serum biomarkers, such as the PRO-3 and ELF scores, are also utilized (13, 14). Although progression of MASH fibrosis was once believed to follow a simple, stepwise “two-hit” model, a “multiple-hit” hypothesis is now favored, implying a much more complex and multifactorial mechanism (15). Notably, the progression of fibrosis is not driven solely by these stromal cells but is regulated by diverse intercellular crosstalk (1, 16). The liver is constantly exposed to bacterial and dietary antigens via the portal circulation and thus has a unique immune environment (17). Under fibrotic conditions, which are characterized by excessive deposition of the extracellular matrix and resultant tissue scarring, injured hepatocytes and sinusoidal endothelial cells damaged by oxidative stress or exogenous antigens interact with immune cells in the liver (Kupffer cells, monocyte-derived macrophages, T cells, etc.) to create an inflammatory, profibrotic microenvironment. Persistent tissue injury and inflammatory stimuli can initiate the release of profibrotic cytokines from hepatocytes, LSECs, and inflammatory immune cells. Profibrogenic cytokines, such as TGF-β1, IL-13, and PDGF, stimulate stellate cells, inducing them to produce collagen and α-smooth muscle actin (α-SMA) (18, 19). Simultaneously, chronic inflammatory signals, accumulated stiff ECM, and hypoxia can enhance the recruitment of immune cells by stellate cells and sinusoidal endothelium (18). Numerous candidates have been explored in drug development for liver fibrosis, particularly MASH, PSC, and PBC (11, 20). Recently, in the field of MASH, THR-β agonists and GLP-1 agonists have been reported to improve fat accumulation, which causes inflammation leading to fibrosis, thereby ameliorating fibrotic conditions (21, 22). However, none of the therapies has succeeded in directly halting or reversing fibrosis. This underscores the need for more comprehensive elucidation of the interactions between stellates and immune cells and of the mechanisms driving fibrogenesis.

### 2.2 New insights into liver fibrosis obtained via single-cell technologies

In liver fibrotic diseases, transcriptomic analyses, such as microarrays and bulk RNA-seq, have been attempted since the year 2000, highlighting the importance of immune/inflammatory pathways and TGF-β1 and ECM production pathways. A breakthrough in single-cell analysis of the human liver came in 2018 when MacParland et al. performed scRNA-seq on healthy donor livers, profiling both parenchymal and non-parenchymal cells and redefining hepatocytes, stellate cells, liver sinusoidal endothelial cells (LSECs), macrophages, T cells, B cells, and other subsets at the transcriptomic level (23). Notably, they found that CD68^+^ liver macrophages comprise at least two subsets: one inflammatory subset expressing LYZ, S100A8/A9, and CD74, and another subset expressing CD5L and MARCO, consistent with the expression in resident Kupffer cells (23). The understanding of the pathology of human liver fibrosis was further propelled by a 2019 study in which Ramachandran et al. applied scRNA-seq to cirrhotic human liver (24). Based on unbiased clustering of single cells from cirrhotic liver tissue, they identified a TREM2-positive macrophage population with a profibrogenic phenotype, termed “scar-associated macrophages” (SAMacs) (24). Employing cell–cell communication analysis enabled by single-cell data, they also detected significant crosstalk between SAMacs and stellate cells. This not only unraveled a novel pathogenic cell type but also underscored the importance of analyzing intercellular interactions in understanding disease mechanisms (24). Following this study, the details of heterogeneous macrophage populations in the liver have rapidly emerged. In another investigation using scRNA-seq of liver from patients with MASH, integrative analysis of single-cell and bulk RNA profiles led to the identification of disease-associated SPP1^+^ macrophages that produce profibrotic factors and correlate with outcomes, such as cirrhosis progression and hepatocellular carcinoma (25). Cell–cell communication analysis indicated active signals related to cell adhesion and actin cytoskeleton regulation between these macrophages and other cells (25). Notably, both SAMacs and SPP1^+^ macrophages highly express FABP5. Based on this shared feature, some researchers group them together as “FABP5 macrophages” (26). In an analysis of cirrhotic livers, FABP5-high macrophages were defined as a distinct subset abundant in patients; these cells showed high expression of fibrogenic mediators (CSF-1, TGF-β1, PDGF, etc.) and, in coculture experiments, promoted fibrosis through interactions with hepatic stellate cells (26).

Single-cell analysis techniques, including approaches that integrate spatial transcriptomics and proteomics, have continued to evolve and provide insights into how different macrophage subtypes arise. For example, inflammatory macrophages located around bile ducts, termed lipid-associated macrophages (LAMs), are regulated by local lipid exposure and are induced in steatotic regions, whereas maintenance of the resident Kupffer cell population depends on crosstalk with stellate cells via the ALK1–BMP10 axis (27). LAMs express TREM2, SPP1, and GPNMB, similar to SAMacs and FABP5^+^ macrophages, which suggests that they may represent overlapping or identical populations (27). Another study reported a CD9^+^TREM2^+^SPP1^+^ macrophage subset that promotes fibrosis by producing TGF-β1 and PDGF through IL-17/STAT1-mediated signals, indicating multiple regulatory inputs into the TREM2^+^SPP1^+^ profibrotic macrophage phenotype (28). The most recent comprehensive study applied scRNA-seq to paired liver and blood samples spanning all disease stages, from metabolic-associated fatty liver to MASH (29). In MASH livers, they observed accumulation of TREM2^+^S100A9^+^ inflammatory macrophages and S100^high^HLA^low^ dendritic cells, forming a distinctive inflammatory niche. The results of cell–cell communication analysis indicated that monocytes (and monocyte-derived cells) are the dominant interactors with stellate cells throughout early to late MASH, whereas interactions between lymphocytes and stellate cells notably peak in early disease and diminish in later stages (29). They also found that inflammatory macrophages and neutrophils predominantly localized near the central veins, whereas regulatory macrophages and NKT cells were enriched in the periportal regions (29). Additionally, severe MASH is associated with an increase in regulatory T cells (Tregs) and myeloid-derived suppressor cells (MDSCs) with impaired T cell function and increased NK cell cytotoxic activity (29). The involvement of innate lymphoid cells (ILCs) in liver fibrosis has also been reported, and scRNA-seq of tissues from patients with human liver cirrhosis revealed the characteristic detection of liver-specific ILCs (30). Notably, group 1 ILCs, which express CD49a, CD94, CD200R1, and CXCR6, exhibit prominent infiltration into fibrotic areas (30). A study in which scRNA-seq was performed on the liver tissue from patients with PBC showed an increase in Th1-like cells, suggesting that they interact with LSECs and HSCs via IL-2, TNF, and IFN-γ (31). An increase in Th1-like cells correlates with the progression of fibrosis and cirrhosis, indicating that these cells contribute to the pathogenesis (31). Single-cell studies have also indicated that chronic liver disease leads to the accumulation of “exhausted” T cells, raising the possibility that these cells might be failing in performing antifibrotic functions (32). Furthermore, liver fibrosis models utilizing human organoids are also being developed. Ouchi et al. created human iPS cell-derived liver organoids that recapitulate key liver-resident cell types, including Kupffer cells and hepatic stellate cells (33). They successfully induced fibrotic responses in these organoids by exposing them to free fatty acids or modeling MASLD-like conditions (33). In this study, scRNA-seq was used to profile each constituent cell in the organoids, confirming that they retain cellular diversity reflective of the human liver (33). More recently, functionally mature organoids with integrated biliary network structures have been reported (34), and further technological advances including cholangitis-based liver fibrosis disease models are anticipated.

Mouse studies have complemented human single-cell findings by allowing for mechanistic probing. Single-cell profiling in mice has similarly identified multiple liver macrophage subtypes. Miyamoto et al. used spatial transcriptomics in the mouse liver to show the phenotypic differences between macrophages near the central veins and those near the portal tracts (35). Periportal macrophages, which are more exposed to gut-derived antigens, exhibit a regulatory phenotype that produces IL-10 and has high MARCO expression (35). Using MARCO-deficient mice, they demonstrated that these periportal macrophages play a protective role—in chronic colitis-induced PSC-like cholangitis/fibrosis and in MCD + high-fat diet-induced MASH, loss of this MARCO^+^ macrophage population led to the exacerbation of inflammation and fibrosis specifically in periportal regions (35). In a murine MASH model examining fibrosis regression, the population of tissue-resident memory CD8^+^ T cells (CD8 Trm) increased during both fibrosis progression and recovery phases. scRNA-seq revealed a Granzyme A^+^ CD8 Trm subset that expanded during the fibrosis resolution phase, and these cells promoted fibrosis reversal by inducing apoptosis of activated stellate cells via the Fas ligand (10, 36). Takimoto et al. performed scRNA-seq analysis of hepatic mononuclear phagocytes during the fibrosis resolution phase in a CCl4-induced mouse liver fibrosis model (37). They revealed that Ly-6C^low^ macrophages, which promoted fibrosis regression via matrix metalloproteinase (MMP) production, highly expressed SPP1, CD44, and TREM2 (37). In this study, TLR4 was also demonstrated to play a crucial role in the induction of Ly-6C^low^ macrophages, and SPP1 and TREM2, known as SAM markers, were also induced in association with TLR4 (37).

**Figure 1:**
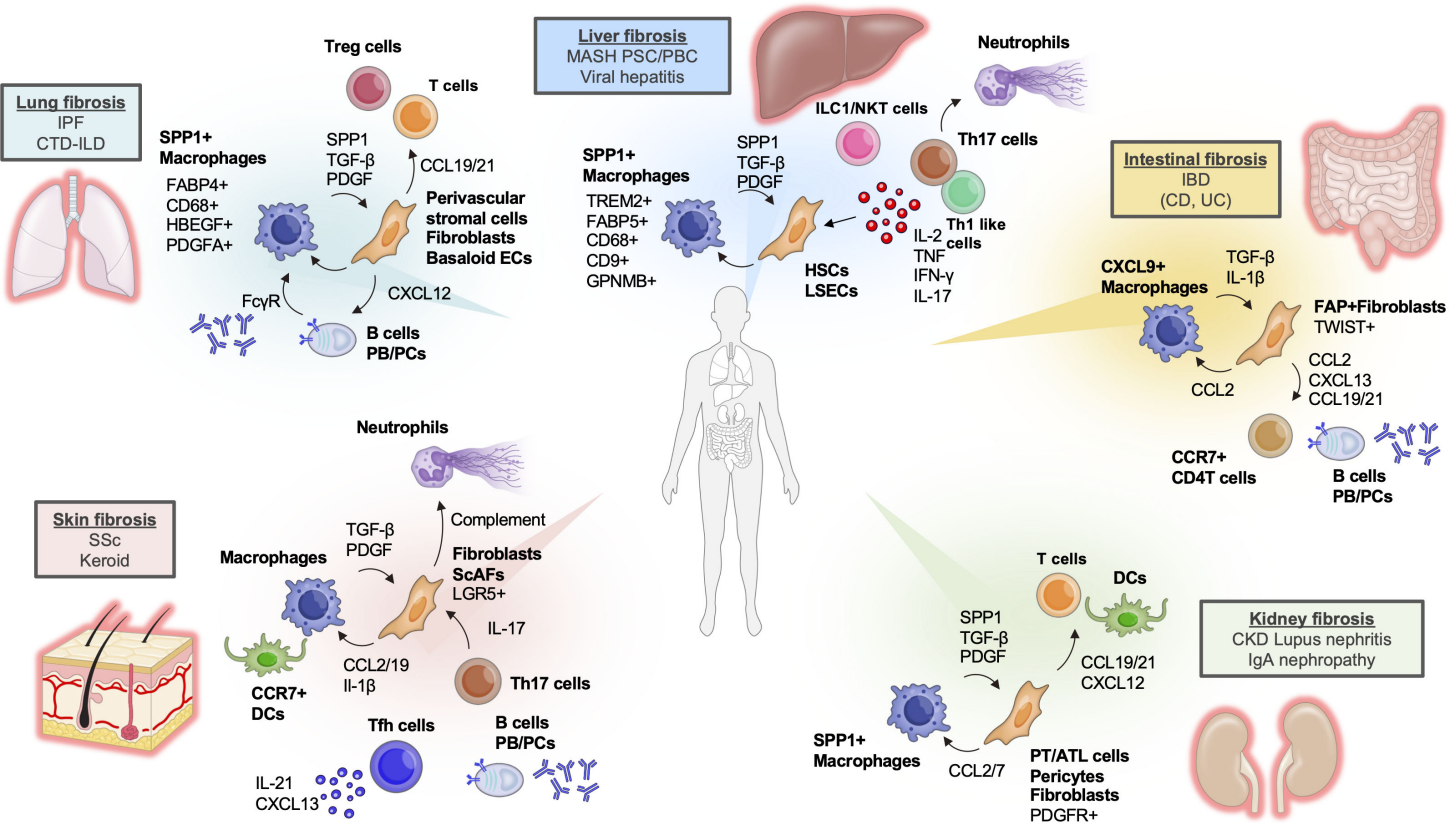
Overview of the immunological mechanisms across human multi-organs revealed by single-cell technologies. In hepatic fibrosis, analyses of liver cirrhosis patients have demonstrated that SPP1^+^TREM2^+^ scar-associated macrophages (SAMs) promote fibrogenesis via interactions with hepatic stellate cells (HSCs) (24). Several reports have also characterized these SAMs by expression of markers such as FABP, CD68, CD9, and GPNMB (25–29). In primary biliary cholangitis (PBC) patients, Th1-like cells interact with HSCs and liver sinusoidal endothelial cells (LSECs) through cytokines including IL-2, TNF, and IFNγ (31). The involvement of innate-like lymphocytes such as NKT cells and ILC1 has also been suggested (29, 30). In pulmonary fibrosis, the significance of SPP1^+^ macrophages has been reported; these cells express FABP4 and CD68 (57). Additionally, HBEGF^+^ macrophages and PDGFA^+^ cells have been found to be characteristic in patients with idiopathic pulmonary fibrosis (IPF) (19). Various parenchymal cells in the lung produce CXCL12 to recruit B cells and plasma cells, and induce macrophage-mediated TGFβ production via FcγR, promoting fibrosis (63). Furthermore, CCL19/21 derived from parenchymal cells contributes to the recruitment of T cells (63). In renal fibrosis, as in the liver and lung, the contribution of SPP1^+^ macrophages has been reported. SPP1^+^ macrophages interact with parenchymal cells such as aPT and aPTC via CCL2/7, promoting fibrogenesis (83). Fibrosis is also induced by myofibroblasts derived from pericytes and fibroblasts expressing PDGFR (85). Stromal cell-derived CXCL12, CCL19, and CCL21 present in the fibrotic region recruit T cells and dendritic cells (DCs), further facilitating fibrosis (86). In skin fibrosis, SSc-specific fibroblasts (ScAFs) have been defined in systemic sclerosis (SSc) patients (105). These disease-specific fibroblasts induce the recruitment and activation of macrophages and DCs via CCL2/CCL19 and IL-1β, and also activate neutrophils through the complement pathway (105). In addition, tissue infiltration of Tfh cells, B cells, and plasma cells producing CXCL13 is observed, suggesting an increasing involvement of the adaptive immune system (103). In intestinal fibrosis, the interaction between CXCL9^+^ macrophages and FAP^+^ fibroblasts has been identified as a key driver of fibrotic pathology (116). FAP^+^ fibroblasts highly expressing TWIST recruit macrophages via CCL2, and CCR7^+^CD4 T cells via CCL19/21 (116). Accumulation of IgG^+^ plasma cells, B cells, phagocytes, and T cells has also been revealed in patients (119). Reports of successful removal of tissue B cells using CAR-T cells suggest an important contribution of B cell lineages (126), which has been relatively overlooked thus far.

In summary, single-cell analysis of the liver has advanced our understanding of heterogeneous macrophage subsets and their interactions with stromal cells, clarifying the key role of coordinated immune–stromal processes in driving liver fibrosis. Further exploration of additional cell types (e.g., various lymphocyte subsets) is expected in future studies. Targeting of the interactions between immune and stromal cells (such as stellate cells) is anticipated to yield novel therapeutic strategies for fibrosis. New single-cell-derived insights for the liver and other organs (described in later sections) are summarized in **Figure 1**.

## 3 Lung fibrosis

### 3.1 Background and general mechanisms of lung fibrosis

Among pulmonary fibrotic diseases, idiopathic pulmonary fibrosis (IPF) is a representative progressive and often fatal condition (38, 39). Fibrosis in IPF involves the accumulation of fibrous tissue in the alveolar walls, leading to progressive stiffening of the lung tissue and irreversible impairment of pulmonary function (40). Pulmonary fibrosis is diagnosed via computed tomography (CT) or pathological examination. However, lung tissue biopsy carries a 1–2% mortality risk. Therefore, the use of biopsy is limited to cases where fibrosis cannot be determined via CT (41, 42). The multi-tyrosine kinase inhibitor nintedanib and the antifibrotic agent pirfenidone are used as approved therapies for IPF. More recently, following FDA approval, the PDE4B inhibitor nerandomilast has also gained considerable attention (43). However, these treatments merely slow the rate of decline in lung function and do not stop disease progression or induce remission (44). Chronic inflammation and immune dysregulation are recognized as important contributors to pulmonary fibrosis. However, the fact that both approved drugs have broad mechanisms of action controlling multiple pathways suggests that this disease requires simultaneous modulation of many signals (44). The pathogenesis of IPF and other lung fibroses is believed to begin with injury to alveolar epithelial cells, followed by infiltration of macrophages, neutrophils, and T cells (40, 45). These immune cells release profibrotic signals that drive fibroblast activation and fibrogenesis (46). Under conditions of chronic inflammation, macrophages produce TGF-β and PDGF, which induce differentiation of lung fibroblasts into myofibroblasts and spur the production and deposition of ECM (47, 48). The contribution of Th2 cytokines to lung fibrosis was recognized relatively early compared with that in other organs: IL-4 and IL-13 can induce lung fibroblasts to produce collagen, whereas IFN-γ (a Th1 cytokine) exhibits antifibrotic effects (19, 46, 49). IL-17A produced by Th17 cells has also been suggested to worsen fibrotic pathology by promoting fibroblast activation (50, 51). Indeed, multiple studies have reported that IPF lungs show a shift toward Th2 and Th17 immune responses compared with healthy lungs, implying that the balance between myeloid cell subsets and CD4^+^ T cell subsets (Th1/Th2/Th17/Treg) is crucial (52–54). The roles of B cells and other lymphocyte populations in lung fibrosis are unclear and remain an area of ongoing investigation (55, 56).

### 3.2 New insights into lung fibrosis obtained via single-cell technologies

Recent single-cell analyses have provided detailed maps of immune cells in pulmonary fibrosis. One important emerging theme is the diversity and centrality of macrophages. Christina Morse et al. performed scRNA-seq on cells from lung tissue from healthy individuals and patients with IPF, sampling both relatively preserved upper lobe and severely fibrotic lower lobe regions in case of the latter (57). They identified three major macrophage populations: monocytes, FABP4^+^ tissue-resident macrophages, and SPP1 (Osteopontin)^+^ macrophages. The SPP1^+^ macrophages were notably expanded in progressive (lower lobe) IPF and were found to colocalize with myofibroblasts, which indicated that they promote disease progression by activating fibroblasts (57). More recently, studies integrating scRNA-seq with spatial transcriptomics revealed spatially resolved pathological niches in IPF (58). In this analysis, eight distinct cellular niches were identified in the lung tissue, each defined by specific cell phenotypes and marker gene signatures (58). In IPF lungs, two particularly disease-relevant niches were described: a “fibrotic niche” around airways consisting of myofibroblasts and aberrant basaloid epithelial cells, and an “airway macrophage niche” adjacent to small airways featuring airway macrophages including SPP1^+^ profibrotic macrophages (58). Unlike classic lymphoid aggregates, these fibrotic niches were often situated around areas of remodeled vasculature (58). Notably, the integrin receptors ITGAV and ITGB6, which bind SPP1, were highly expressed on aberrant basaloid epithelial cells, indicating that SPP1-mediated immune–stromal interactions occur specifically in fibrotic lesions (58). The aberrant basaloid cells also showed evidence of the activation of WNT signaling, supporting the notion that a combination of these pathological events (aberrant epithelial activation, profibrotic macrophages, and myofibroblasts) drives IPF, providing insights that have only recently been made possible with the use of single-cell and spatial transcriptomic approaches (58). Consistent with these findings, other groups have observed enrichment of SPP1^+^CD68^+^ macrophages in the fibrotic “honeycomb” lesions of IPF lungs (57). Similar transcriptional signatures of these profibrotic macrophages have been reported in multiple single-cell studies on IPF lungs, reinforcing the understanding about their key roles in disease pathogenesis (59–61). In other reports, HBEGF+ macrophages and PDGFA+ macrophages have been identified as disease-specific macrophages through analyses of the lungs of patients with IPF (19). Besides tissue analysis, studies on bronchoalveolar lavage fluid (BALF) in pulmonary fibrosis have provided insights into the immune environment, with less-invasive sampling. For example, in a comprehensive study using single-cell RNA-seq analysis of BALF from various CTD-ILDs, such as SS, DM, RA, SSc, and AAV, unbiased data suggesting differences in lung pathology for each CTD, which had not been well characterized earlier, were obtained (62). In SS-derived ILD, activation of B cells and the adaptive immune system in BALF was suggested, whereas in DM, features such as IFN and other virus responses, and in RA, an increase in neutrophils, became apparent (62). These findings indicate that the mechanisms leading to fibrosis in CTDs may differ depending on the underlying disease.

In addition to macrophages, single-cell studies have revealed new facets of other immune cells involved in lung fibrosis. Yang et al. recently used spatial transcriptomics in IPF lungs and revealed an abnormal accumulation of plasma cells at fibrotic loci. Healthy lungs contain few or no plasma cells, which is a notable observation (63). They also identified a subset of lung fibroblasts with high CXCL12 expression in IPF tissue, which appeared to attract CXCR4^+^ plasma cells, thereby creating a niche enriched in plasma cells (63). Additionally, they discovered a novel perivascular stromal cell subset producing CCR7 ligands (CCL19/21) that recruit T cells to fibrotic areas (63). The plasma cells within the fibrotic niche were suggested to contribute to the exacerbation of fibrosis by inducing the production of TGF-β from alveolar macrophages via Fcγ receptor signaling (63). In contrast, another study showed that in patients with IPF, the proportions of naïve and memory B cells in the lung tissue were increased, whereas those of plasmablasts were decreased, and that aberrant B-cell receptor features in naïve B cells might contribute to disease progression (64). These seemingly divergent findings indicate that the role of B-cell lineage in human IPF is complex and requires further study. The role of regulatory T cells (Tregs) in IPF has also been reported (65). Unterman et al. profiled immune cells from the lungs and peripheral blood of patients with IPF via scRNA-seq. Notably, in patients with progressive, advancing IPF, they found that most lymphocyte populations were numerically decreased compared with that in controls or in patients with more stable IPF, except for classical monocytes and Tregs, which were increased in both the blood and lungs. Patients with higher Treg abundance had poorer outcomes, indicating that the accumulation of immunosuppressive Tregs in fibrotic lungs might paradoxically contribute to disease progression, possibly by forming fibrotic niches in concert with monocytes, which were concurrently expanded. Similarly, another group observed Treg enrichment in IPF lungs, although they did not report major shifts in other T-cell subsets (61). These findings highlight a potential pathogenic role of Tregs (normally anti-inflammatory cells) in the fibrotic lung environment. Organoid-based fibrosis models have also been reported for the lung. Co-culturing fibroblasts derived from fibrotic lung tissues with AT2 organoids induced a morphological transition from the typical grape-like structures to cystic forms and led to a marked increase in MUC5B expression (66). Overexpression of MUC5B is a hallmark abnormality in human IPF lungs, and this model was shown to recapitulate c key features of human fibrotic lung pathology. In this study, trajectory analysis based on single-cell data revealed that fibroblasts drive the differentiation of AT2 cells into MUC5B-expressing AT2-like cells. Future technological advances are expected to yield lung fibrosis organoid models that also incorporate immune-mediated components of disease.

Results from studies using animal models align with human data and offer mechanistic insights. Single-cell profiling of bleomycin-injured mouse lungs has underscored the importance of SPP1^+^ monocyte-derived macrophages in lung fibrosis (67). These macrophages produce SPP1, TGF-β1, and PDGF and interact with lung fibroblasts to promote fibrosis in the bleomycin model (67). Other studies showed an accumulation of TREM2^+^ monocyte/macrophages in bleomycin-treated mouse lungs, and that genetic deletion or pharmacological blockade of TREM2 reduced fibrosis (68, 69). A comparative analysis of immune cells in human IPF and the bleomycin mouse model found that key genes of the SPP1^+^ macrophage (such as SPP1, CD68, and APOE) are induced in both human and mouse fibrotic lungs (69). In a study that analyzed both the fibrotic progression and resolution phases of the bleomycin model using single-cell and spatial transcriptomics (70), CSMD1^+^ fibroblasts with ECM-secreting properties were induced during the progression phase, whereas CD248^+^ fibroblasts with tissue repair-promoting properties were induced during the resolution phase (70). The authors further identified an inflammatory and ECM-producing niche composed predominantly of adventitial fibroblasts, immune cells such as NK and T cells, and vascular endothelial cells, as well as a pro-resolving niche centered on alveolar and peribronchial fibroblasts characterized by strong fibroblast–fibroblast interactions (70). Importantly, similar fibroblast subtypes and spatially organized niches were also observed in lung tissues from patients with IPF (70). Additionally, depletion of plasmablasts in a mouse bleomycin model attenuated lung fibrosis, lending support to the pathogenic importance of B-lineage cells observed in human studies (71). However, significant differences exist between mouse models and human IPF. For example, in the mouse pulmonary fibrosis model induced by silica, marked activation of CD4^+^ and CD8^+^ T-cell subsets occurs in the alveolar microenvironment; however, this phenomenon does not necessarily coincide with what is observed in human IPF (61). These results highlight the need to develop new models that better recapitulate human fibrotic pathology to meet the unmet medical need for antifibrotic drugs.

Overall, these cutting-edge findings collectively demonstrate that lung fibrosis is not merely a fibroblast-driven disorder but is heavily orchestrated by immune cells. This paradigm shift opens avenues for new treatments. For instance, targeting specific pathological immune cells or their signals (such as fibrogenic plasma cells, profibrotic T-cell subsets, or key macrophage pathways) could lead to novel therapies for suppressing or even reversing pulmonary fibrosis.

## 4 Kidney fibrosis

### 4.1 Immunological mechanisms of kidney fibrosis

In the kidneys, chronic inflammation due to underlying conditions, such as diabetes, hypertension, and autoimmune nephritis, leads to interstitial fibrosis and glomerulosclerosis, driving the progression of chronic kidney disease (CKD) (72). In fibrotic kidneys, normal tissue structure and function are disrupted, and collagen deposition in the interstitium impairs the filtration capacity of the organ (73). Fibrosis secondary to CKD is a common pathological process associated with progressive renal diseases. Although pathological evaluation remains the gold standard for assessing renal fibrosis, other indicators such as DWI-MRI have also been proposed (74, 75). Therapies, such as renin–angiotensin system inhibitors and SGLT2 inhibitors, can slow CKD progression in type 2 diabetes; however, no treatment exists for reversing or curing established renal fibrosis (76–78). When nephrons are injured, tubular epithelial and glomerular cells release chemokines and damage-associated molecular patterns (DAMPs), which recruit macrophages, neutrophils, and T cells into the kidney (79). After infiltrating, immune cells, such as macrophages, T cells, and dendritic cells, secrete profibrotic and inflammatory mediators (TGF-β, FGF, PDGF, IL-1β, TNF-α, etc.), activating renal fibroblasts and podocytes to become myofibroblasts (72, 80). Consequently, a pathological loop is established in which persistent fibrotic tissue stress maintains chronic inflammation, thereby perpetuating the progression of fibrosis. Circulating monocytes have been suggested to migrate into the kidney and, under local inflammatory or fibrotic cues, differentiate into profibrotic macrophages that promote fibrosis (81, 82). However, as numerous immune cell types contribute to renal fibrosis and exert diverse context-dependent functions, capturing the full complexity of this process without bias is difficult using traditional analyses.

### 4.2 New insights into kidney fibrosis obtained via single-cell technologies

Single-cell analyses comparing healthy and CKD kidneys have begun to clarify immune cell composition and phenotypic changes in renal fibrosis. For example, an scRNA-seq study of healthy versus CKD human kidneys revealed CKD-specific immune alterations (83). CD16^+^ NK cells (known to have renoprotective roles) were found to be diminished in CKD kidneys, whereas CCR7^+^ dendritic cells and naïve CD4^+^ T cells (associated with inflammation) were increased (83). Similar to other organs, the injured kidneys also showed an accumulation of SPP1^+^ macrophages in fibrotic areas (83). Analysis of cell–cell communication in this study revealed interactions between SPP1^+^ macrophages and parenchymal cells, namely adaptive proximal tubule (aPT) cells and adaptive ascending thin limb (aATL) cells, via CCL2/CCL7 chemokine signaling, suggesting a pathogenic role for these SPP1^+^ macrophages in CKD progression (83). In IgA nephropathy, single-cell RNA-seq of the kidney tissue showed that mesangial cells in diseased glomeruli produce SPP1 and engaged in crosstalk with macrophages via CCL2, whereas CD8^+^ T cells in the kidney exhibited reduced expression of effector molecules, such as granzymes, implying that they might be functionally impaired (84).

Other groups have also performed scRNA-seq using kidney tissues from patients with CKD and identified periostin-positive myofibroblasts that produce high levels of ECM (85). These cells were found to express PDGFR and were shown to originate from pericytes and fibroblasts (85). Injured tubular epithelial cells express JAG1, which activates Notch signaling in adjacent fibroblasts. Macrophages secrete PDGF-BB, stimulating PDGF receptors on fibroblasts and promoting their activation (85). These findings highlight the cell–cell interactions involved in disease pathology. Furthermore, NKD2 was identified as a marker specifically expressed by these cells (85). More recently, an ambitious multi-omics study integrated single-cell, single-nucleus (snRNA-seq), spatial transcriptomics, and single-nucleus ATAC-seq to profile healthy and diseased human kidneys, including samples from patients with obesity- or hypertension-related kidney disease (86). This comprehensive atlas cataloged the cell types in the kidney. In this study, by combining spatial information, the kidney microenvironment was classified into four niches, one of which was a fibrotic niche characterized by dense aggregates of immune cells and fibroblasts (86). In these fibrotic regions, stromal cells were found to secrete chemokines, such as CXCL12, CCL19, and CCL21, which possibly contribute to the recruitment of T and dendritic cells (86). Conversely, immune cells and injured epithelial cells present in the niche strongly expressed profibrotic signals, such as PDGF-BB and TGF-β1, which activate fibroblasts (86). This study also identified two distinctive subtypes of injured proximal tubule cells (iPT cells), one with high VCAM1 expression and the other with high KIM-1 expression. Notably, KIM-1^high^ iPT cells were dramatically expanded in samples from patients with CKD and were concentrated within the fibrotic areas. These KIM-1^high^ iPT cells highly expressed profibrotic factors, such as SPP1 and PDGFB, strongly suggesting that they actively interact with and stimulate fibroblasts (86). Furthermore, the authors derived a “fibrotic niche signature” and showed that patients whose kidney biopsies had high fibrotic niche scores were at significantly greater risk of decline in renal function, emphasizing the clinical significance of these immune–stromal microenvironments.

The roles of lymphocytes and other nonmyeloid cells in renal fibrosis have also been elucidated using mouse models. Do Valle Duraes et al. applied scRNA-seq to two mouse models of renal ischemia-reperfusion injury to examine the changes in immune cells during fibrosis (87). They observed the most prominent changes in tissue-resident macrophages and T cells, including an expansion of IL-33^+^ IL-2Rα^+^ tissue Tregs, which were suggested to have protective functions (87). In another study, Doke et al. used a unilateral ureteral obstruction (UUO) model to perform comprehensive single-cell analysis and discovered unexpected crosstalk between basophils and injured renal tubule cells (88). They found that CXCL1 produced by profibrotic tubule cells recruited CXCR2^+^ basophils into the kidney and that basophil depletion ameliorated fibrosis in UUO mice (88). In a rat model of hyperuricemic nephropathy, scRNA-seq similarly identified pathogenic interactions, particularly CCL3/4–CCR5 signaling, between proximal tubule epithelial cells and macrophages, which are critical for establishing the fibrotic microenvironment, and highlighted epithelial cell-derived factors (e.g., Hsbp1 and Cldn4) to be considerably upregulated profibrotic mediators (89).

In a study using a mouse model of renal injury induced by unilateral ischemia-reperfusion injury, the transition from AKI to CKD was successfully recapitulated (90). In this study, the kidney tissues were collected from the disease model at five different time points, and scRNA-seq and spatial transcriptomics were performed. The results revealed that, in the acute phase, damaged tubular epithelial cells and macrophages were colocalized, whereas in the chronic phase, macrophages were positioned in close proximity to fibroblasts (90). A novel subset of macrophages interacting with fibroblasts, termed ECM Remodeling-Associated Macrophages (EAMs), was identified. These EAMs were derived from monocytes, and they were demonstrated to interact with fibroblasts via IGF signaling, thereby promoting renal fibrosis (90).

Collectively, these findings indicate that renal fibrosis is an actively immune-driven process. Immune cells, such as macrophages and T cells, undergo phenotypic changes in CKD kidneys and engage in pathogenic crosstalk with epithelial cells and fibroblasts.

## 5 Skin fibrosis

### 5.1 Immunological mechanisms of skin fibrosis

Skin fibrosis, observed in conditions, such as systemic sclerosis (scleroderma), keloids, and hypertrophic scars, involves excessive ECM accumulation in the dermis (91, 92). Hyperactivated dermal fibroblasts produce and deposit collagen, leading to skin hardening or raised scar tissue (91, 92). In clinical practice, assessment using the Rodnan Skin Score based on skin thickness measurement is common, whereas skin biopsy is most often used for research purposes such as omics analysis (93, 94). In systemic sclerosis (SSc), a systemic autoimmune disease, widespread fibrosis occurs with a major contribution from adaptive immunity (T and B cells) (45). In contrast, local innate immune-driven inflammation triggered by injury is believed to play a leading role in the development of keloids and hypertrophic scars (95). In all cases, interactions between the immune system and tissue stromal cells are considered to be crucial in disease development. In injured skin, macrophages and T cells infiltrate and release growth factors and cytokines, such as TGF-β and IL-13, which stimulate fibroblasts (96, 97). Activated dermal fibroblasts secrete chemokines that recruit and retain additional immune cells, thereby sustaining chronic inflammation (45). The skin has long been recognized for a strong involvement of the IL-17/Th17 axis in fibrotic processes, more than any other organ (98). IL-17 produced by Th17 cells can induce the proliferation of dermal fibroblasts and production of collagen, thereby promoting fibrosis (98). However, until recently, the precise contribution of Th17 cells to skin fibrosis and its contrast with fibrotic mechanisms in other organs were not clearly defined. In SSc, B cell-targeted therapy (rituximab, an anti-CD20 antibody) has shown efficacy in some clinical trials, underscoring a significant role for B cells in driving fibrosis. However, the detailed involvement of B-lineage cells in fibrotic skin remains insufficiently understood (99).

### 5.2 New insights into skin fibrosis obtained via single-cell technologies

Th17 cells play important roles in various skin conditions. For example, IL-17A neutralizing antibodies are highly effective in treating psoriasis (100). Single-cell studies have reinforced the evidence of their importance in fibrotic skin diseases. In a scRNA-seq study of human keloid lesions versus normal scar tissue, Deng et al. identified at least four transcriptionally distinct fibroblast subsets in the dermis (101). A “mesenchymal fibroblast” subset was dramatically expanded in keloid scars compared with that in normal scars and was characterized by high ECM production (101). Notably, the same subset was also increased in SSc skin, indicating that it represents a common profibrotic fibroblast phenotype that can emerge under different skin fibrosis conditions (101). Another single-cell study on keloid skin reported a marked increase in Th17 cells in the lesions, accompanied by the activation of macrophages and type 2 conventional dendritic cells (cDC2) (102). Th17 cells were suggested to contribute to keloid pathology by secreting IL-17A, which drives collagen production by fibroblasts and local fibroblast accumulation. In another study, a detailed analysis of T-cell profiles in patients with SSc and healthy individuals was performed using scRNA-seq (103). Subsets of circulating and tissue-resident T cells were present in both healthy and SSc skin samples. Many cell clusters exhibited common gene expression patterns in both healthy subjects and patients; however, a CXCL13^+^ T-cell cluster specific to the SSc skin was identified (103). These CXCL13^+^ T cells displayed a gene expression profile similar to that of Tfh cells, suggesting that they may promote B-cell responses within the skin (103). Another study demonstrated the importance of B cells using scRNA-seq. In a 3D skin model, B cells derived from patients with SSc were found to infiltrate the skin more prominently than those derived from healthy individuals. Furthermore, scRNA-seq revealed that clusters of B cells infiltrating the tissue exhibited activated and antibody-producing profiles (104). The diversity of fibroblasts in SSc and the functions of each fibroblast subtype are also being gradually elucidated. In 2022, a large integrated single-cell transcriptomic and epigenomic (scATAC-seq) analysis of patients with SSc profiled immune and stromal cells in blood and skin lesions (105). This study identified a novel fibroblast subset, scleroderma-associated fibroblasts (ScAFs), defined by the expression of the stem cell marker LGR5 (105). ScAFs were present in both healthy and SSc skin. In healthy skin, they apparently serve as a homeostatic fibroblast population, expressing not only ECM genes, but also MMPs, complement-regulatory proteins, and angiogenic factors, indicating a central role in maintaining tissue homeostasis. However, in SSc skin, the frequency of ScAFs was significantly reduced, and they showed a shift toward a profibrotic, activated phenotype with high AP-1 activity and increased production of ECM components (105). The analysis also revealed that, in SSc, ScAFs interact with immune cells via multiple pathways—they can suppress NK- and CD8 T-cell activity via HLA-E, activate macrophages via IL-1β, and activate macrophages and neutrophils via complement pathways (105). These findings suggest that ScAFs may normally help regulate immune responses and tissue integrity and that their loss or phenotypic change in SSc removes a break in fibrosis while adding further profibrotic signals. Other studies have highlighted the diversity of fibroblasts. Clark et al. cultured tissue samples obtained via skin biopsy from healthy individuals and patients with SScs (106). They classified fibroblasts that spontaneously migrated out of skin explants as migratory fibroblasts, and those remaining in the tissue as resident fibroblasts (106). The motile fibroblasts exhibited strongly induced TGF-β signaling and expressed CCN5 and MMP2, whereas the resident fibroblasts expressed relatively weaker TGF-β signaling but characteristically expressed C7, CCL19, and CCL2 (106). Resident fibroblasts were more abundant in the later phases of disease pathology (106).

In a mouse model, single-cell analysis of bleomycin-induced dermal fibrosis revealed accumulation of TREM2^+^ macrophages in the fibrotic skin (107). Notably, depletion of TREM2^+^ macrophages in this model led to worsening of fibrosis, which indicated that, unlike in many other contexts, these macrophages might have an antifibrotic, inflammation-resolving function in the skin (107). Macrophages can adopt phenotypes that dampen inflammation and fibrosis, for instance, via efferocytosis (clearing apoptotic cells) and production of anti-inflammatory cytokines (107). The TREM2^+^ macrophages observed in the fibrotic skin might represent such a phenotype that aids in limiting fibrosis. In a recent study, single-cell transcriptome and surface proteome analyses (CITE-seq) were performed on untreated patients with SSc to investigate the relationship between immune cells and progression of organ damage (108). The results showed that, in SSc patients presenting with renal crisis, EGR1^+^ CD14^+^ monocytes were significantly increased in those with SRC, and in renal tissue, these cells differentiated into THBS1^+^ macrophages that accumulated at sites of injury (108). In contrast, in patients with SSc presenting with interstitial lung disease (ILD), a subset of CD8^+^ T cells exhibited a type II interferon signature and was found at a high frequency in the peripheral blood and lung tissue of patients with progressive ILD (108). These findings revealed diverse immunological features associated with organ manifestations linked to SSc. Overall, these findings indicate that skin fibrosis is tightly linked to immune cells, particularly Th17 cells and macrophages. This provides mechanistic support for the current therapeutic efforts in fibrotic skin diseases aimed at targeting the Th17/IL-17 pathway (and possibly other immune targets).

## 6 Intestinal fibrosis

### 6.1 Immunological mechanisms of intestinal fibrosis

Intestinal fibrosis is a serious complication of chronic inflammatory bowel diseases (IBD) (109). Persistent inflammation can chronically injure the intestinal wall, leading to excessive collagen deposition and thickening of the bowel wall, resulting in fibrotic strictures (109). Intestinal fibrosis is typically evaluated by the degree of fibrosis observed in surgical histopathology, whereas cross-sectional imaging used for detecting stricture cannot accurately determine the extent of fibrosis (110, 111). More than half of the patients with Crohn’s disease develop such strictures over the long term, and in many cases, surgical resection is required because of the lack of antifibrotic medication (112). Intestinal fibrosis is believed to occur partly because certain immune responses associated with tissue remodeling remain abnormally active even after the overt inflammation subsides. The gut wall contains networks of fibroblasts, smooth muscle cells, and other mesenchymal cells that orchestrate wound healing (109). However, under chronic inflammatory conditions, the myofibroblasts (the key fibrogenic effector cells) are persistently activated, with pathways, such as TGF-β, IL-13, IL-17A, and PAI-I, driving their activation (113). In IBD, immune cells, including macrophages, T cells, and innate lymphoid cells, release not only these fibrogenic cytokines but also inflammatory cytokines, such as IL-1β, IL-33, IL-36, and TNF-α, which further stimulate fibroblasts (113). Conversely, activated fibroblasts secrete chemokines, such as CXCL12, CCL2, CXCL1, and CXCL8, to recruit additional immune cells (114, 115). Thus, a self-reinforcing loop of inflammation and fibrosis is established in the intestinal tissue, such that even after the primary inflammation resolves, fibrosis continues autonomously. The complexity of the intestinal structure and cell composition made it challenging for earlier methods to identify the specific cells and signals responsible for this process.

### 6.2 New insights into intestinal fibrosis obtained via single-cell technologies

Single-cell analyses are currently used to identify previously unrecognized mesenchymal cell subsets that drive intestinal fibrosis. Zhang et al. performed scRNA-seq on fibrotic stricture lesions from patients with Crohn’s disease and discovered a subset of fibroblast activation protein (FAP)-positive fibroblasts as the key pathogenic cells promoting intestinal fibrosis (116). FAP^+^ fibroblasts were dramatically expanded in fibrotic regions compared with that in non-fibrotic areas and expressed high levels of ECM genes and the profibrotic transcription factor TWIST1. Analysis of cell–cell interaction revealed that CXCL9^+^ inflammatory macrophages communicate with FAP^+^ fibroblasts via IL-1β and TGF-β signaling, thereby driving fibroblast activation and fibrosis in Crohn’s disease strictures (116). Single-cell and spatial analyses have also illumined the broader cellular context of fibrotic intestinal niches (117). Kong et al. combined scRNA-seq and spatial transcriptomics to examine stenotic ileal lesions in Crohn’s disease and identified 68 distinct cell clusters (117). They observed a clear increase in IgG-secreting plasma cells in tissues from the stricture site compared with that in controls, indicating robust local antibody production in fibrotic lesions (117). They also noted an accumulation of CCR7-high CD4^+^ T cells, and that the fibrotic lesions contained lymphoid follicle-like aggregates of B and T cells, suggesting that local B–T cell interactions (possibly via the CCR7–CCL19/21 axis) contribute to fibrosis. Furthermore, they classified fibroblasts into two subsets: one with a high collagen-producing capacity and another “inflammatory” subset that produced several inflammatory cytokines (117). Notably, they showed that fibrotic strictures are not sustained by fibroblasts and immune cells alone but also involve other cell types, such as interstitial cells of Cajal and enteric neurons intermingled within the fibrotic strictures, suggesting that the fibrotic niche is a complex multicellular network (117). Current IBD therapies mainly target inflammation; however, these single-cell findings indicate that targeting key genes or pathways in these complex cellular interactions (for example, signals between macrophages and fibroblasts or factors produced by pathological fibroblast subsets) could offer new antifibrotic treatment approaches.

In another report, more than 60 intestinal tissue samples (from fibrotic sites, nonfibrotic sites, and healthy controls) collected from patients with Crohn’s disease were analyzed via scRNA-seq and spatial transcriptomics, allowing for an integrated evaluation of spatial cellular arrangements and gene expression (118). A fibroblast subset specifically present at fibrotic sites, which exhibited high expression of chemokines, such as CCL2 and CXCL13, as well as activation markers, was discovered (118). These inflammatory fibroblasts were suggested to interact with immune cells and to activate TNF responses and antigen presentation pathways (118). The expression of antigen presentation capabilities and costimulatory molecules was also indicated, suggesting that these cells may have functions similar to those of synovial fibroblasts in rheumatoid arthritis (118). The mechanisms underlying resistance to TNF treatment in patients with Crohn’s disease have also been analyzed using single-cell technologies. Martin et al. used scRNA-seq and CyTOF to analyze lesional and non-lesional tissues from the ileum, as well as peripheral blood from patients with Crohn’s disease, and elucidated the molecular and cellular background of treatment resistance (119). They identified a pathological cell module called GIMATS (IgG plasma cells, inflammatory mononuclear phagocytes, activated T cells, and stromal cells) within inflammatory lesions (119). This module was specifically present in patients who did not respond to anti-TNF therapy. Cell–cell interactions revealed that inflammatory macrophages and mature dendritic cells played central roles within the GIMATS module, and the presence of this module correlated with the failure of anti-TNF therapy (119). Organoid models that capture features of intestinal fibrosis have also been developed using intestinal organoids. In a study employing scRNA-seq to investigate disease-associated molecular signatures in rectal organoids derived from patients with Crohn’s disease (120), the patient-derived organoids exhibited abnormalities in metabolic, epigenetic, and proliferative pathways but lacked inflammatory signaling (120). These findings underscore the necessity of incorporating immune–epithelial crosstalk to more faithfully model intestinal fibrotic disease.

Mouse models have provided some parallel insights. For instance, Jasso et al. performed a single-cell analysis on rat and mouse colitis models. scRNA-seq of lamina propria stromal cells revealed more than six fibroblast clusters and fibroblast populations that changed in an inflammation-dependent manner including IL-11-producing fibroblasts (121). Another group that performed scRNA-seq in mouse chronic DSS colitis models identified FAP^+^ fibroblasts and CXCL9^+^ macrophages as key players in fibrosis (122). Furthermore, Wang et al. focused on Tregs and reported a pathway through which Treg-derived AREG exacerbated intestinal fibrosis (123). In a chronic DSS colitis model using AREG-deficient mice, inflammation was more severe than that in wild-type mice, whereas fibrosis remained mild (123). Additionally, in experiments transferring either wild-type or Areg-deficient Tregs into immunodeficient mice, fibrosis was significantly suppressed in the group receiving Areg-deficient Tregs (123).

The emerging single-cell evidence helps explain why purely anti-inflammatory therapy often fails to completely prevent fibrosis in Crohn’s disease. Once a profibrotic fibroblast population like FAP^+^ fibroblasts becomes established and gets engaged in mutual reinforcement with immune cells such as CXCL9^+^ macrophages, fibrosis may progress even if gut inflammation is controlled. Therefore, identifying these culprit cells and their interactions provides concrete targets for intervention and suggests that effective antifibrotic therapy will require breaking of local immune–stromal feedback loops.

## 7 Treatment concepts for fibrotic diseases unveiled via single-cell technologies

Fibrotic diseases lack effective disease-modifying therapies and represent a substantial unmet medical need. In some fibrotic conditions such as IPF and scleroderma-related ILD, antifibrotic drugs (pirfenidone, nintedanib) have been approved. Recently, the PDE4B inhibitor nerandomilast was also approved by the FDA for treating IPF and ILD, increasing the therapeutic options available for pulmonary fibrosis (43). In CKD, agents, such as RAS inhibitors and SGLT2 inhibitors, are helpful to some extent; In MASH (metabolic steatohepatitis), new drugs such as the THR-β agonist resmetirom and the GLP-1 agonist semaglutide have shown efficacy and exhibit some antifibrotic effect (21, 22). However, these options predominantly slow the disease progression but do not usually lead to true reversal or cure of fibrosis. Moreover, their mechanisms often broadly involve modulating multiple pathways or treating upstream metabolic or inflammatory drivers rather than directly targeting specific fibrotic mechanisms. The importance of understanding fibrosis biology in detail is evident. Numerous therapeutic concepts based on prior knowledge (TGF-β inhibitors, ASK1 inhibitors, PPAR agonists, FXR agonists, etc.) have been tested, but none have led to approved antifibrotic drugs, as of date (3).

**Figure 2:**
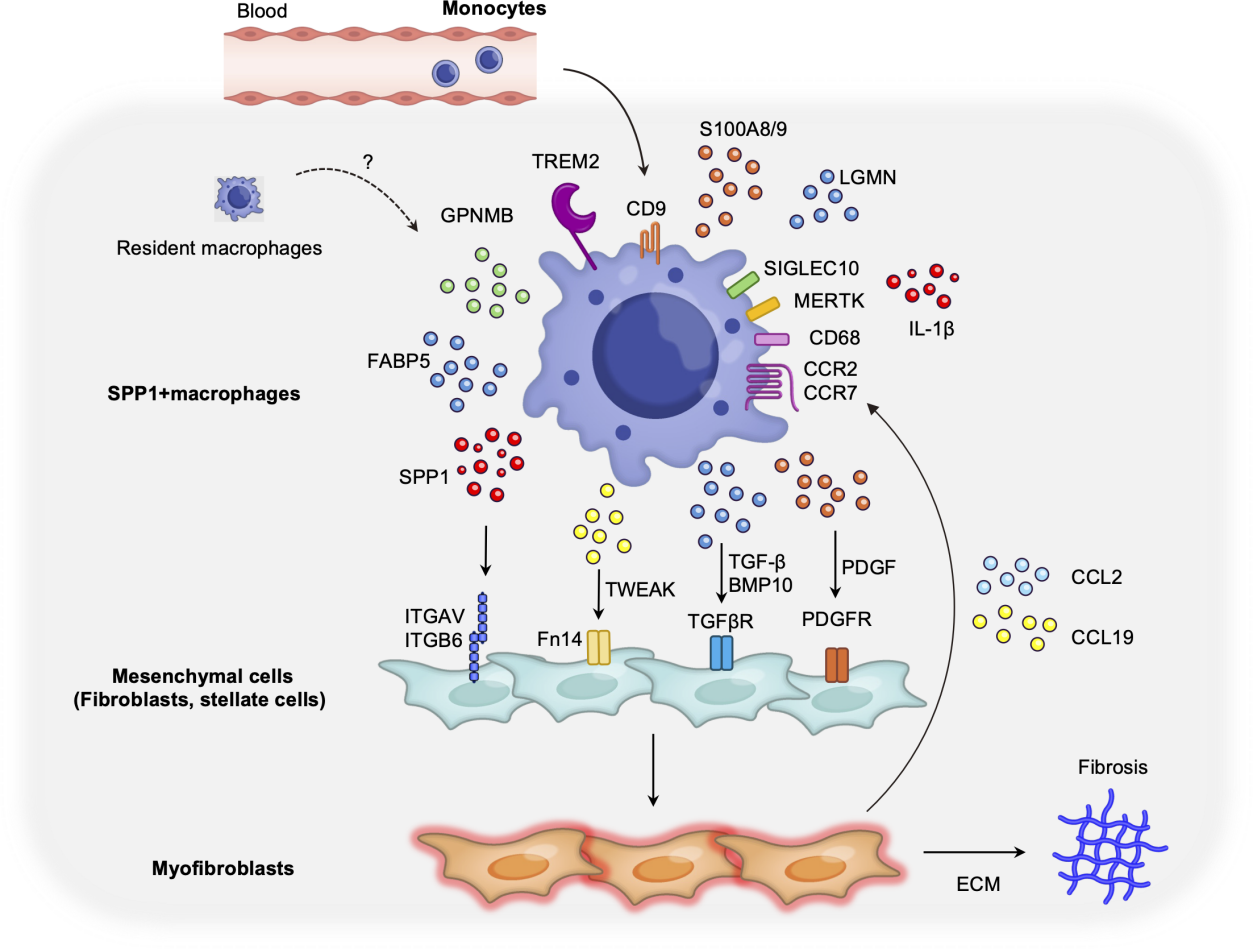
Newly characterized pro-fibrotic SPP1^+^ macrophages in human pathological tissues identified by single-cell technologies. Myeloid cells, in contrast to lymphoid lineages, often display ambiguous marker expression based on surface antigens, implying substantial heterogeneity and, until recently, a lack of clear classification. Advances in single-cell analysis have enabled the identification of secreted factor expression at single-cell resolution, leading to a redefinition of SPP1^+^ macrophages as key pro-fibrotic cells in fibrotic disease states in multi-organs (24, 130–132). TREM2 and CD9 are core markers that are commonly expressed in SPP1^+^ macrophages across all organs. Molecular mechanisms have been deciphered for the liver and lungs wherein SPP1^+^ macrophages promote fibrosis by interacting with parenchymal cells via signaling pathways, such as PDGF and TWEAK (28). Although the molecular mechanisms for SPP1+ macrophages in other organs such as the kidney have not been elucidated to the same extent as in the liver or lungs, it is believed that in each case, they interact with fibroblasts and epithelial cells within fibrotic lesions and promote collagen deposition via profibrotic signaling such as TGF-β and PDGF (131). Liver SPP1^+^ macrophages typically express CD68, GPNMB, FABP5, IL1B, and S100A8/9 (25–29). In the lung, SPP1^+^ macrophages have been reported to express CD68 as well as MERTK, LGMN, SIGLEC10, and MMP9 (28, 57). A recent study also demonstrated that intestinal SPP1^+^ macrophages express S100A8/9 (133). Although the precise origins of SPP1^+^ macrophages—whether they arise from circulating monocytes or from tissue-resident macrophages—remain unclear, current evidence suggests that in the liver and lung they are predominantly derived from circulating monocytes (1, 24, 28, 131). Parenchymal cells stimulated by SPP1^+^ macrophages become activated myofibroblasts, directly inducing fibrosis through ECM production, and serving as a central hub by secreting chemokines that recruit additional macrophages, thus perpetuating a pathogenic loop. Spatial analyses have revealed that these cells are predominantly located within the fibrotic regions, indicating their pivotal role as the origin of pathology.

Novel insights from single-cell and spatial omics are now bringing us closer to the core of fibrotic pathophysiology and are expected to inform new therapeutic strategies. For example, TREM2^+^/SPP1^+^ scar-associated macrophages were a previously unrecognized myeloid subset that could not be easily delineated using classical surface markers; scRNA-seq enabled their discovery, and strikingly similar populations have been reported from the fibrotic niches of multiple organs (24, 26, 57, 58, 67, 83). The characteristics of the profibrotic SPP1-positive macrophages that exist across organs are summarized in **Figure 2**. Because they appear in diverse fibrotic diseases, therapies targeting these macrophages may have broad applications and are highly promising. A key issue in formulating such strategies is the identification of the best approach for targeting these cells. Potential approaches include inhibitory antibodies or small molecules against integrin receptors used by these macrophages, or conversely, the development of agents (e.g., antibodies and CAR T cells) that selectively eliminate cells expressing markers, such as TREM2 and CD9. Achieving selectivity is a challenge, and identifying more specific surface markers or using combination targeting (bi-specific or tri-specific antibodies) may be required to avoid off-target effects.

Single-cell studies have also highlighted the contribution of B and plasma cells to fibrotic diseases, such as IPF and SSc (63, 104). In SSc, B-cell depletion therapy (rituximab, anti-CD20) has been effective in some cases, and it may be worthwhile to explore B cell–targeted treatments for IPF where they have not been fully tested (124). Moreover, a recent understanding of immunology suggests that the depth and breadth of B-cell elimination are important. Traditional anti-CD20 therapy primarily depletes circulating and lymphoid B cells, but newer approaches such as anti-CD19 (targeting a broader range of B lineage cells) or the concept of “deep depletion” that also clears tissue-resident B cells are gaining attention (125). Indeed, extensive depletion of B and plasma cells (for example, using CAR T cells or T cell-engaging bispecific antibodies against B cell markers) could potentially yield stronger and more durable antifibrotic effects in diseases, such as IPF and SSc, if these cells are truly pathogenic. Recently, although investigations have been conducted in only a very limited number of cases of UC, there have been reports of remarkable efficacy in multidrug-resistant patients using B cell-targeted therapy with CD19 CAR T-Cell Therapy (126). The involvement of B cells in UC remains unclear; however, Fabian Müller et al. showed local analysis using single-cell RNA sequencing has suggested the involvement of B cells (126). These findings strongly indicate the importance of understanding the local pathology with single-cell technologies and controlling pathogenic cells at the local level.

Pathogenic fibroblast subsets have also emerged as potential therapeutic targets. For instance, FAP^+^ fibroblasts in intestinal fibrosis, LGR5^+^ ScAFs in SSc, and analogous subsets in other organs could be therapeutic candidates (105, 116). For aiming at elimination or modulation of fibroblasts in fibrotic tissues, the issue of marker specificity arises. Fibroblast markers such as FAP are fairly broadly expressed on activated fibroblasts including some involved in normal wound healing; therefore, simply targeting all FAP^+^ cells with a CAR T cell or monoclonal antibody could be associated with risks. Innovative targeting strategies might be needed to home in on the truly pathogenic fibroblasts, perhaps using combinations of markers or local delivery systems. One intriguing approach specific to liver fibrosis is the use of vitamin A-coupled liposomes loaded with drugs, which are taken up by hepatic stellate cells owing to their natural vitamin A storage function, thereby delivering therapy selectively to fibrogenic cells in the liver (127, 128). Although this method is organ-specific, it exemplifies how leveraging unique cell biology can add specificity. Analogous strategies might also be developed for other organs. Another approach is to target the molecular drivers within these cells. Single-cell studies have identified new transcription factors and signaling molecules associated with fibrosis, such as TWIST1 in intestinal fibroblasts and AP-1 and other pathways in ScAFs (105, 116). These can be attacked with precision medicines, such as antisense oligonucleotides or RNA interference. The expansion of therapeutic modalities beyond traditional small molecules and monoclonal antibodies indicates that we now have tools, such as bispecific antibodies, CAR T cells, therapeutic vaccines, and siRNAs, at our disposal.

Although immune cells are involved in the occurrence of fibrosis, broad immunosuppressive therapies have not been sufficiently effective against established fibrosis. This may stem from the fact that, once fibrotic cascades are activated, feed-forward loops are established between immune cells and fibroblasts, making each compartment partially self-sustaining. Consequently, transiently modulating only one side of this circuit is often insufficient. The observation that only broad-spectrum agents—such as pirfenidone, nintedanib, and nerandomilast—which act on both immune and stromal compartments, have shown therapeutic benefit further supports this notion. In addition, immune cells implicated in fibrosis, including SPP1^+^ macrophages, acquire distinct profibrotic phenotypes that may not be adequately targeted by conventional anti-inflammatory drugs. Therefore, improving fibrotic pathologies will require therapeutic strategies that appropriately modulate these newly characterized immune populations identified through single-cell analyses. In this context, the challenge will be to deploy treatments in combinations or creative ways to, for instance, concurrently hit multiple fibrotic drivers but mainly within the fibrotic tissue to minimize systemic side effects. Future antifibrotic regimens might conceivably involve combination therapies, perhaps simultaneously blocking a key growth factor, depleting scar-associated immune cells, and reprogramming fibroblasts, tailored to each patient’s fibrotic profile. It outlines representative cells and factors involved in fibrosis (e.g. TREM2^+^ macrophages, Th17 cells, pathogenic plasma cells, FAP^+^ fibroblasts, CD248^+^ fibroblasts, TGF-β, IL-13, IL-17A, etc.) and potential targeted therapies corresponding to each (such as macrophage recruitment inhibitors, anti-cytokine or anti-integrin therapies, B-cell depletion or plasma-cell targeting, antifibrotic CAR T cells, etc.). This evolving paradigm involves controlling multiple crucial pathways simultaneously while conferring tissue specificity to reduce adverse effects. Single-cell discoveries provide a blueprint for what to target, and advances in drug development provide new ways to target them.

## 8 Conclusions and future perspectives

Single-cell and other omics technologies have advanced rapidly over the recent years, enabling integrated multi-omics (transcriptomic, chromatin, proteomic, and spatial data) analyses of even minute human tissue samples. These approaches now allow us to obtain high-resolution, unbiased clinical evidence. They have been proven to be especially powerful for profiling tissue myeloid cells and fibroblasts, which were difficult to categorize using conventional surface markers, leading to the identification of a stream of new cell subsets. In the past, investigating the interactions between multiple cell types in the same patient sample was challenging, forcing researchers to gather fragmentary data. With multi-omics data from single samples and sophisticated cell–cell interaction analyses, the unbiased understanding of disease mechanisms has advanced. The ability to incorporate spatial information has been a particularly important development, and it is now possible to pinpoint which immune–stromal interactions are truly operative in the specific microenvironment of diseased lesions, greatly aiding the pathological insight and therapeutic development.

As we move towards leveraging these insights for treatment, one prospect is to develop more spatially targeted or cell type-specific interventions. Therefore, achieving tissue-specific cell death or inactivation has become increasingly important. For example, to target a pathogenic immune–stromal interaction that occurs in a certain niche, one might need modalities that act preferentially at that site, such as locally delivered therapies or systemically administered cells that home into the fibrotic tissue. In addition, to use multi-omics findings for patient stratification or diagnostics, complex signatures must be distilled into a few accessible biomarkers. Methods for finding single markers or small panels that capture the essence of a multi-omics signature are expected to mature. Alternatively, if single-cell profiling becomes sufficiently fast, affordable, and standardized, these technologies can be used directly in clinical settings for detailed patient profiling and personalized therapy decisions.

Despite the progress afforded by single-cell technologies, some areas require further advancement. Multi-omics studies with large patient cohorts or longitudinal sampling, although being undertaken, have still been scarce. These will be necessary to understand the variability and temporal dynamics of fibrosis, for instance, in comparing early *vs*. advanced disease or progressive *vs*. regressing fibrosis in patients. Notably, fibrosis in both humans and animals is reversible to some extent, and these reversal processes likely involve intricate immune–parenchymal coordination, such as the CD8 Trm or Ly-6C^low^ macrophage-mediated resolution in the liver (36, 129). Studying fibrosis regression in humans via multi-omics could reveal critical pathways for therapeutic stimulation. Additionally, applying multi-omics to biopsies from patients who do not respond to current therapies may aid in pinpoint alternative fibrotic pathways that these therapies fail to hit, thereby guiding next-generation treatments.

Integration and comparison of human data with those from animal models will continue to be invaluable. Animal models allow genetic and interventional experiments that are not possible in humans, helping in the validation of hypotheses, such as in confirming the function of a cell subset by depletion or in testing a new treatment concept. Nonetheless, it is clear that no single animal model perfectly replicates human fibrosis for every disease; therefore, comparative multi-omics can highlight where models align or diverge, and new models may need to be developed for poorly represented aspects, such as models capturing the role of adaptive immunity in fibrosis. It should also be noted that, beyond macrophages—which have attracted substantial attention—many roles of other immune cell types, including novel subsets of T cells, B cells, and ILCs, likely contribute to fibrosis but remain largely uncharacterized.

In conclusion, the confluence of rapidly improving single-cell, spatial, and integrative omics technologies with innovative therapeutic modalities from small molecules to engineered cells offers unprecedented opportunities to tackle fibrotic diseases. However, because single-cell analyses alone are anticipated to fully elucidate the core mechanisms of the pathology or to provide definitive evidence. A close integration of immunology, genomics, bioinformatics, and clinical research is indeed essential for developing safe and effective antifibrotic therapies based on the increasingly detailed cellular insights obtained through these approaches. Continued interdisciplinary efforts are needed to translate detailed cellular insights into safe and effective antifibrotic therapies. In the coming years, these efforts are expected to yield breakthroughs in treating, and perhaps even reversing, fibrosis in various organs.
